# Removal of Cadmium and Lead from Tires Discarded in the Open Sea with Multicomponent Nanoparticles from Sugarcane Bagasse

**DOI:** 10.3390/nano15221700

**Published:** 2025-11-10

**Authors:** Erika Murgueitio-Herrera, Pablo Carpio, Paola Bungacho, Luis Tipán Tapia, Christian Camacho, Alexis Debut

**Affiliations:** 1 Centro de Nanociencia y Nanotecnología, Universidad de las Fuerzas Armadas ESPE, Av. Gral. Rumiñahui s/n, Sangolquí P.O. Box 171-5-231B, Ecuador; apdebut@espe.edu.ec; 2 Departamento de Ciencias de la Vida y de la Agricultura, Carrera Ingeniería en Biotecnología, Universidad de las Fuerzas Armadas ESPE—Sede Santo Domingo de los Tsáchilas, Vía Santo Domingo—Vía Quevedo Km. 24 Hda. Zoila Luz Avenida Quevedo, Santo Domingo P.O. Box 3-703-914, Ecuador; pacarpio1@espe.edu.ec (P.C.); pabungacho@espe.edu.ec (P.B.); cocamacho1@espe.edu.ec (C.C.); 3 Departamento de Ciencias Administrativas y Económica, Universidad de las Fuerzas Armadas ESPE, Av. Gral. Rumiñahui s/n, Sangolquí P.O. Box 171-5-231B, Ecuador; latipan@espe.edu.ec

**Keywords:** nanoparticles, cadmium, lead

## Abstract

This study addresses the environmental challenge of end-of-life tire accumulation, a major source of toxic metals such as lead and cadmium in marine ecosystems. As a sustainable solution, multicomponent metal-oxide nanoparticles (Fe_3_O_4_, ZnO, CaO, MgO, and minor CaCO_3_) were green-synthesized from sugarcane bagasse and stabilized with blackberry (*Rubus glaucus*) extract. Structural characterization (XRD, SEM, TEM, and EDS) confirmed their crystalline inorganic composition. Pb^2+^ was almost completely removed (95–99%) within 15–30 min using 50–100 mg of nanoparticles, with ~80–90% efficiency at 75 mg. Cd^2+^ removal showed dose-dependent kinetics: ~90% removal occurred within 10 min at 75 mg, while 50 and 100 mg reached ~60–70% after 60 min. Equilibrium, kinetic, and thermodynamic analyses revealed that Pb^2+^ adsorption followed the Langmuir model (R^2^ = 0.982) with monolayer chemisorption, whereas Cd^2+^ obeyed the Freundlich model (R^2^ = 0.945), indicating heterogeneous multilayer adsorption. Pb^2+^ removal fitted a pseudo-second-order model (R^2^ = 0.991), while Cd^2+^ followed a pseudo-first-order behavior (R^2^ = 0.958). Thermodynamic parameters (ΔG° < 0, ΔH° > 0, ΔS° > 0) confirmed a spontaneous and endothermic process. Sugarcane-bagasse-derived Fe_3_O_4_–ZnO–CaO–MgO nanomaterials act as sustainable and effective adsorbents for marine heavy metal removal.

## 1. Introduction

The planet is facing an acute environmental crisis driven by the overexploitation of natural resources, with end-of-life tires (ELTs) emerging as one of the most challenging solid wastes due to their slow degradation rate, high disposal costs, and significant landfill requirements [1,2,3]. In developed regions such as the European Union and the United States, ELTs are typically subjected to recycling operations, including shredding and material recovery [4]. However, in many Latin American countries, these wastes are often discarded in rivers, lagoons, or directly into the sea, causing serious environmental, social, and economic consequences [5]. In Ecuador, initiatives such as the 2019 agreement between the Ministry of Environment and SEGINUS have sought to strengthen integrated waste management systems [6]; nevertheless, the environmental risks associated with improper tire disposal remain largely underestimated [7].

A major environmental concern associated with end-of-life tires (ELTs) is the release of heavy metals—particularly lead (Pb), cadmium (Cd), and zinc (Zn)—due to their high toxicity and bioaccumulative nature [8,9,10]. Numerous studies have reported the leaching of Pb, Cd, and Zn from tire residues in landfills and aquatic environments, contributing significantly to soil and water contamination [9,10]. Cadmium, a highly mobile and carcinogenic element, has been detected in marine systems at concentrations ranging from <5 to 110 ng L^−1^ [11,12,13,14], whereas lead, a persistent neurotoxin, occurs in seawater at approximately 10^−3^ mg kg^−1^ [15,16,17]. Both metals are known to bioaccumulate through the marine food web, posing severe ecological and human health risks [18,19,20,21,22,23].

Conventional remediation techniques, including physical and chemical treatments, are often costly, energy-intensive, and may generate secondary toxic byproducts [24,25,26,27,28]. Consequently, sustainable alternatives such as bioadsorption using agricultural residues, phytoremediation, and bioremediation have gained attention as environmentally friendly and cost-effective strategies [29,30,31,32].

In addition to heavy metals, end-of-life tires (ELTs) contribute to marine pollution through the release of microplastics and tire wear particles (TWP), also known as crumb rubber granulate (CRG). Recent studies have shown that TWP and CRG release zinc (Zn) at ecotoxicologically relevant concentrations, with leaching dynamics strongly influenced by salinity and exposure time [33,34]. Comprehensive reviews further indicate that TWP constitute a significant fraction of coastal and oceanic microplastic pollution, acting as carriers of heavy metals and persistent organic pollutants, thereby posing potential risks to marine ecosystems and human health [35].

Tires are primarily composed of natural and synthetic rubbers, carbon black, silica, and various chemical additives, including zinc oxide (ZnO, ~1–2 wt%), which represents the main intentionally added metal [33,34]. Lead (Pb) and cadmium (Cd), although not deliberately incorporated, occur as trace contaminants originating from pigments and stabilizers, with reported concentrations ranging from 0.1–2 mg kg^−1^ and 0.5–10 mg kg^−1^, respectively [35]. Despite their lower abundance compared to Zn, Cd is highly mobile and carcinogenic, while Pb is a persistent neurotoxin that tends to accumulate in sediments [36].

This issue is particularly critical along the Ecuadorian coast, where discarded tires of unknown origin are frequently found in marine environments. Although this practice is unauthorized, it continues to occur, thereby increasing the risk of Cd and Pb leaching into coastal ecosystems and their subsequent bioaccumulation within marine biota.

Nanotechnology offers an innovative platform for heavy metal remediation, as metal oxide nanoparticles exhibit high adsorption capacity, strong selectivity, and tunable physicochemical properties suitable for environmental applications [37,38]. In particular, green synthesis using biological precursors provides a sustainable alternative by avoiding toxic reagents and simultaneously adding value to agricultural byproducts [39,40]. Previous studies have demonstrated the effectiveness of iron oxide nanoparticles synthesized from natural extracts such as clove and coffee in the adsorption of Cd^2+^ and Ni^2+^, while also revealing notable antimicrobial properties [41]. Among iron oxides, maghemite (γ-Fe_2_O_3_) and magnetite (Fe_3_O_4_) possess distinctive size-dependent magnetic characteristics, making them ideal candidates for advanced technological and environmental applications [42,43,44].

Sugarcane bagasse (*Saccharum officinarum*), the most abundant byproduct of the sugar industry, is rich in minerals such as silica, calcium, iron, magnesium, and zinc [45,46,47]. This composition makes it a valuable and renewable precursor for nanoparticle synthesis. Previous studies have utilized sugarcane bagasse to produce magnetic nanoparticles with superparamagnetic properties for oil recovery applications [48]. However, despite significant advances in green nanotechnology, evidence of its direct application in environmental remediation remains scarce [49,50].

This study investigates the potential of biogenic nanoparticles synthesized from sugarcane bagasse for the removal of Pb^2+^ and Cd^2+^ leached from discarded tires in the marine environment of the Galápagos Islands. Nanoparticles of iron oxide (FeO), zinc oxide (ZnO), calcium oxide (CaO), calcium carbonate (CaCO_3_), and magnesium oxide (MgO) were synthesized, characterized, and evaluated for their adsorption capacity in seawater. The findings demonstrate that sugarcane bagasse, an abundant and low-cost industrial byproduct, can serve as a sustainable precursor for producing highly efficient nanoparticles for heavy metal removal. This approach not only aligns with the principles of the circular economy but also contributes to the preservation of fragile marine ecosystems, providing a green and scalable solution for mitigating heavy metal contamination.

## 2. Materials and Methods

### 2.1. Study Area

This research was conducted under laboratory conditions at the Advanced Materials Laboratory of the Nanoscience and Nanotechnology Center (CENCINAT) of the Universidad de las Fuerzas Armadas (ESPE). The research center is located at the main campus in Sangolquí, Ecuador (0°18′53″ S, 78°26′36″ W), in the inter-Andean valley of north-central Ecuador, at an altitude of 2748 m above sea level. The laboratory maintains an average temperature of 25 °C.

### 2.2. Chemicals

Reagents for water analysis were purchased from USA Hach Company P.O. Box 389 Loveland, Colorado. For the purification of the synthesis products and the preparation of the reaction solutions, ultrapure water from a Milli-Q system (Conductivity 0.28 µS/cm, Millipore, Direct Q, Darmstadt, Germany) was used, while the ethanol used was absolute grade, as with ACS ISO (Isopropyl Alcohol) 1728 Batch 19.022.310. It was used for the digestion of sugarcane bagasse: NaOH Merck KGaA, CAS Nro 1310-73-2 (Darmstadt, Germany); H_2_O_2_ 30% (*w*/*w*) 0639 BATCH 19555201. Scharlah S.L.

### 2.3. The Extract Black Berry (Rubus glaucus) Preparation

The *Extract mora* (*Rubus glaucus*) were obtained from a local market in Conocoto parish (Quito, Ecuador) and washed with water and neutral soap. Stems, leaves, and flowers were cut into small pieces. Extraction was performed according to previously described methodologies [51,52], using 96% ethanol (Scharlau) at 40% concentration in a 2:1 (*v*/*w*) ratio under rotational agitation. The mixture was sonicated for 10 min with a Branson 3510 ultrasonic device (40% amplitude), filtered through filter paper and then through a 0.45 μm membrane. The solvent was removed with a Yamato RE801 rotary evaporator (Yamato Scientific Co., Ltd., Tokyo, Japan), and the extract was dried at 40 °C for 24 h in a Wiseven oven.

Total phenolic content was determined following the method described by Dewanto et al. (2002) [53], with modifications from Murgueitio et al. (2021) [54], using gallic acid, sodium carbonate, and Folin–Ciocalteu reagent. Absorbance was measured at 760 nm in a Genesys 10 uv UV-Vis spectrophotometer (Thermo Fisher Scientific, Waltham, MA, USA), and results were expressed as mg gallic acid equivalents per liter (mg GAE/L).

Antioxidant capacity was evaluated by the DPPH method [55], using DPPH reagent (SIGMA) and 80% methanol (Merck KgaA). The final solution was sonicated for 20 min in a Cole-Parmer ultrasonic sonicator, and absorbance was recorded with a Perkin Elmer Lambda 35 UV-Vis spectrophotometer (Perkin Elmer, Waltham, MA, USA).

### 2.4. Preparation of Sugarcane Bagasse

Sugarcane bagasse was collected at the Wholesale Market of Machachi (0°30′58″ S, 78°34′01″ W; Mejía Canton, Pichincha Province, Ecuador). Samples were thoroughly washed with water and detergent, and subsequently disinfected to remove organic and inorganic impurities. The cleaned material was dried in a forced convection oven (Memmert SN30, Schwabach, Germany) at 105 °C for 24 h, ground to a fine powder, and subjected to an additional drying step (105 °C, 2 h) to eliminate residual moisture. The dried powder was washed with ultrapure water (Milli-Q^®^, Sigma Aldrich, Santa Clara, CA, USA), oven-dried again (105 °C, 24 h), and sieved to obtain a homogeneous particle size distribution.

Alkaline microwave-assisted digestion was performed using a Multiwave PRO system (Anton Paar, Graz, Austria) with a mixture of NaOH 1 M and hydrogen peroxide (H_2_O_2_, 30%) at a ratio of 1:10:10 (*w*/*v*/*v*). The samples, previously weighed on an analytical balance (Cole Palmer 1000016, Vernon Hills, IL, USA), were placed in digestion vessels. Microwave parameters were set as follows: maximum digestion temperature 240 °C, cooling temperature 30 °C, pressure 100 bar, heating time 10 min, digestion time 15 min, and cooling time 2 h 30 min. After digestion, the solution was filtered to remove undigested residues, yielding a liquid matrix free of organic matter.

The resulting solution was oven-dried at 105 °C for 24 h, and the solid fraction was divided into two portions: one calcined at 500 °C and the other at 900 °C, for 20 min each. Both solids were subsequently washed with Milli-Q^®^ water, sonicated at 40 kHz for 30 min (5 min on/5 min off cycles), and centrifuged. The sonication–centrifugation process was repeated three times. Finally, the solids were resuspended in Milli-Q^®^ water, filtered through 450 µm and 250 µm membranes, oven-dried at 105 °C for 2 h, and stored in a desiccator until use.

### 2.5. Synthesis of Nanoparticles (NPs)

**Figure 1:**
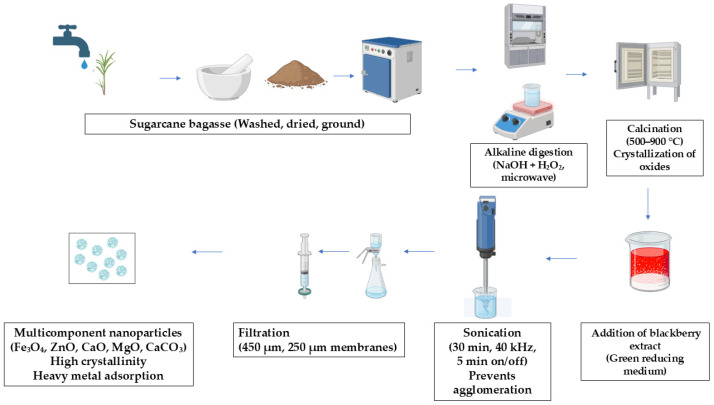
Synthesis of multicomponent nanoparticles.

The synthesis of multicomponent nanoparticles was carried out following the procedure described by Murgueitio et al. (2024) [51]. Initially, sugarcane bagasse was washed, dried, and ground to obtain a clean precursor. The biomass was then subjected to alkaline digestion with NaOH and H_2_O_2_ under microwave-assisted conditions to remove residual organic matter and release inorganic constituents. The resulting solid was subsequently calcined at 500–900 °C to promote the crystallization of metal oxides. To provide a green reducing medium and enhance nanoparticle stability, blackberry (*Rubus glaucus*) extract was incorporated, followed by sonication for 30 min at 40 kHz (5 min on/5 min off cycles) to facilitate dispersion and prevent agglomeration. The suspension was filtered through 450 µm and 250 µm membranes, yielding multicomponent nanoparticles composed of Fe_3_O_4_, ZnO, CaO, MgO, and CaCO_3_. These nanoparticles exhibited high crystallinity and strong potential for heavy metal adsorption in aqueous systems (Figure 1).

### 2.6. Metal Readings from Sugarcane Bagasse

Metal characterization was conducted using Atomic Absorption Spectrophotometry (AAS) with an Analyst 800 instrument (PerkinElmer, Shelton, CT, USA). The equipment was calibrated with standard solutions of known concentrations corresponding to the target metals detected in sugarcane bagasse. A specific hollow cathode lamp was employed for each element (Fe, Zn, Mg, and Ca), following Method 3111 B (direct air–acetylene flame) [56].

The analytical parameters were as follows: iron (Fe), λ = 248 nm, air–acetylene flame, sensitivity 0.12 mg L^−1^, detection limit (DL) 0.02 mg L^−1^; zinc (Zn), λ = 213.9 nm, air–acetylene flame, sensitivity 0.02 mg L^−1^, DL 0.005 mg L^−1^; magnesium (Mg), λ = 285.2 nm, air–acetylene flame, sensitivity 0.007 mg L^−1^, DL 0.0005 mg L^−1^; and calcium (Ca), λ = 422.7 nm, air–acetylene flame, sensitivity 0.08 mg L^−1^, DL 0.003 mg L^−1^.

Prepared samples were introduced into the spectrophotometer using an automatic injection system, and each sample was analyzed in triplicate to ensure analytical precision and reproducibility of the results.

### 2.7. Lead and Cadmium Analysis of Tire Samples

Shredded and sieved tire samples were immersed in seawater at 20 °C and maintained under continuous agitation for 30 days using a Siemens rotary shaker (Model 12023, Munich, Germany). After the exposure period, the liquid phase was filtered and analyzed for lead (Pb) and cadmium (Cd) concentrations by Atomic Absorption Spectrophotometry (AAS), following Method 3111 B (direct air–acetylene flame) [56].

The analytical parameters were as follows: cadmium (Cd), λ = 228.8 nm, air–acetylene flame, sensitivity 0.025 mg L^−1^, detection limit (DL) 0.002 mg L^−1^; and lead (Pb), λ = 283.3 nm, air–acetylene flame, sensitivity 0.5 mg L^−1^, DL 0.05 mg L^−1^.

### 2.8. Materials and Equipment Used in the Characterization of NPs

Nanoparticles (NPs) were characterized to determine their average size, morphology, and crystalline composition. Particle size distribution was first analyzed using a HORIBA LB-550 submicron particle analyzer (HORIBA, Kyoto, Japan), connected to a computer via a SCSI interface and operated with HORIBA LB-550 software (v.3.57; 1996–2005). Measurements were based on the principle of dynamic light scattering (DLS), covering a detection range of 0.001–6 µm (1–6000 nm). The instrument was annually aligned and calibrated with NIST-certified latex standards (monodisperse and polydisperse polystyrene, 20 ± 2 nm and 100 ± 2 nm), with a maximum accepted tolerance of 5%. Repeatability and reproducibility were verified to ensure data accuracy.

Particle morphology and complementary size evaluation were performed by transmission electron microscopy (TEM). A drop of the colloidal suspension was deposited onto carbon-coated copper grids and analyzed using a FEI Tecnai G2 Spirit Twin microscope (FEI, Eindhoven, The Netherlands) operated at 80 kV. Calibration was conducted using gold nanoparticle diffraction, considering an interplanar spacing of 0.24 nm.

The crystalline structure of the NPs was determined by X-ray diffraction (XRD) on an EMPYREAN diffractometer (PANalytical, Almelo, The Netherlands) using Cu Kα radiation (λ = 1.54 Å) in a 2θ configuration. Particle size distribution results were further corroborated using the HORIBA LB-550 analyzer [57].

Surface morphology was examined by scanning electron microscopy (SEM). Samples were coated with a conductive carbon layer using a Quorum Q150R coater (Quorum Technologies, Lewes, UK) and observed under a Tescan Mira 3 microscope (Brno, Czech Republic). Routine maintenance, alignment, and calibration were performed annually to ensure measurement reliability.

### 2.9. Removal of Lead and Cadmium

A specific volume of 50 mL of seawater containing tire leachates was transferred into individual Falcon tubes. The pH was adjusted to 6 using 1 M hydrochloric acid (HCl), and three different doses of the synthesized multicomponent nanoparticles (50, 75, and 100 mg) were added. The suspensions were allowed to react for 3 h, after which the supernatant from each tube was collected for lead (Pb) and cadmium (Cd) quantification by atomic absorption spectrophotometry (AAS). Each metal concentration was measured in triplicate, and the results were expressed as the mean of the three replicates.

Statistical analyses were conducted using InfoStat^®^ software (v.2020; Universidad Nacional de Córdoba, Argentina). Analysis of variance (ANOVA) was applied to evaluate significant differences among treatments, and post hoc multiple comparison tests were performed where applicable. Statistical significance was considered at *p* < 0.05.

Additionally, adsorption equilibrium, kinetic, and thermodynamic studies were conducted to better understand the metal-removal mechanisms. For equilibrium analysis, adsorption isotherms (Langmuir and Freundlich models) were fitted using the residual concentrations of Pb^2+^ and Cd^2+^ obtained at different dosages. Kinetic behavior was evaluated using pseudo-first-order and pseudo-second-order models based on contact time (10–60 min). Thermodynamic parameters (ΔG°, ΔH°, and ΔS°) were calculated from adsorption data at different temperatures (293–323 K) using the van’t Hoff relationship to determine the spontaneity and energy changes in the process.

## 3. Results

### 3.1. Antioxidant Capacity and Polyphenol Concentration

The antioxidant capacity of blackberry extract, determined by the DPPH assay, was 50 ± 2%, with a total polyphenol content of 40 ± 5 mg per 100 g fresh weight (FW). These values are consistent with those reported for Ecuadorian fruits by Vasco et al. [58,59]. In their study, blackberries exhibited among the highest antioxidant capacities and phenolic contents, comparable to native Andean fruits such as mortiño (wild blueberry) and naranjilla, and significantly higher than those of tropical fruits such as banana and papaya [59]. This confirms that blackberry is one of the fruits with the greatest antioxidant potential in Ecuador, supporting its relevance as a natural source of bioactive compounds for nutraceutical and food applications.

In parallel, the alkaline digestion of sugarcane bagasse showed high efficiency in decomposing non-cellulosic components, particularly lignin and hemicellulose. The use of a microwave-assisted digester facilitated both the breakdown and mineralization of solid biomass, which is essential for subsequent elemental analysis by spectroscopic techniques. Compared with dilute sulfuric acid pretreatment—widely reported in the literature (e.g., Canilha et al. [60])—microwave-assisted alkaline digestion with NaOH and H_2_O_2_ achieved comparable or superior results in terms of cellulose accessibility, while providing a faster and more efficient process. Furthermore, this method reduces equipment corrosion, minimizes the formation of inhibitory byproducts such as furfural and hydroxymethylfurfural [61], enhances oxidative delignification through the action of hydrogen peroxide [62], and decreases both chemical consumption and energy demand, making it a sustainable alternative for biomass pretreatment [63].

### 3.2. Analysis of Metals in Sugarcane Bagasse

The concentrations of metals determined in sugarcane bagasse were as follows: iron (Fe), 1.78 ± 0.06 mg L^−1^; zinc (Zn), 19.33 ± 0.02 mg L^−1^; calcium (Ca), 66.06 ± 0.20 mg L^−1^; and magnesium (Mg), 18.10 ± 0.20 mg L^−1^. Reported metal contents in sugarcane bagasse from other studies range from approximately 327 mg kg^−1^ to 2060 mg kg^−1^, depending on the material’s origin and processing conditions [64,65]. The consistency in the order of magnitude—considering the differences between dry-matter and aqueous-extract analyses—supports the reliability of our measurements and confirms the validity of the analytical methodology applied.

### 3.3. Concentration and Removal of Lead and Cadmium in Contaminated Aquatic Systems

Rotor agitation proved effective in reducing lead (Pb) and cadmium (Cd) concentrations in contaminated solutions, reaching 0.40 ± 0.01 mg L^−1^ and 48.7 ± 0.1 mg L^−1^, respectively. This mechanical technique enhances the interaction between metal ions and the remediation medium, thereby improving mass transfer and adsorption efficiency [66]. Compared with other approaches such as biochar adsorption or microalgae biosorption, as reported by Bouida et al. (2022) [67], rotor agitation represents a mechanized and efficient alternative for heavy metal removal. The application of this technique could play a key role in mitigating the adverse effects of toxic metals such as Pb and Cd on human health and the environment, which are associated with renal dysfunction, neurological damage, and ecological imbalance [66,67].

In marine environments, the leaching of heavy metals from discarded tires typically shows zinc (Zn) as the predominant element; however, Pb and Cd, though present at lower concentrations (≤0.01 mg L^−1^), pose greater ecological risks due to their high toxicity (Halsband et al., 2020) [33]. Similar observations in artificial seawater confirmed Pb release at trace levels (Page et al., 2022) [68], and recent reviews have highlighted the recurrent presence of Pb and Cd in tire wear leachates, generally ranging between 10^−3^ and 10^−2^ mg L^−1^ (Frontiers in Marine Science, 2025) [69].

Additionally, Praipipat et al. (2023) [70] synthesized and characterized four sugarcane bagasse-derived adsorbents: bagasse powder (SB), iron (III) oxyhydroxide-doped bagasse powder (SBF), bagasse beads (SBB), and iron (III) oxyhydroxide-doped bagasse beads (SBFB). Experimental results demonstrated that SBFB achieved the highest Pb removal efficiency (100%) at an initial concentration of 10 mg L^−1^ and pH 5, with a minimum dosage of 0.1 g and a contact time of 2 h. These findings confirm the strong potential of modified sugarcane bagasse as an effective and sustainable adsorbent for the remediation of heavy metal-contaminated aquatic systems.

### 3.4. Characterization of Multicomponent Nanoparticles

Dynamic Light Scattering (DLS) analysis of the multicomponent nanoparticles revealed an average diameter of 9.3 nm at a concentration of 0.1 mol/L, in agreement with the properties reported by Lim et al. (2013) [71]. Based on size distribution data obtained by DLS, the Z-average diameters were 16.9 ± 5.2 nm, 21.1 ± 5.5 nm, and 43.1 ± 14.9 nm across different measurements, reflecting the variability typical of colloidal systems. These values confirm that the hydrodynamic size measured by DLS—including the solvation layer—is consistent with the expected range for these nanoparticles, ensuring colloidal stability without significant aggregation.

Dynamic Light Scattering (DLS) analysis of the multicomponent nanoparticles revealed an average particle diameter of 9.3 nm at a concentration of 0.1 mol L^−1^, consistent with the properties reported by Lim et al. (2013) [71]. Based on size distribution data obtained by DLS, the Z-average diameters were 16.9 ± 5.2 nm, 21.1 ± 5.5 nm, and 43.1 ± 14.9 nm across different measurements, reflecting the inherent variability of colloidal systems. These values confirm that the hydrodynamic sizes determined by DLS—including the solvation layer—fall within the expected range for these nanoparticles, indicating excellent colloidal stability and the absence of significant aggregation.

Recent advances emphasize the pivotal role of DLS in correlating nanoparticle size distribution with adsorption performance toward toxic metals. For instance, Bagbi et al. (2017) [72] synthesized iron oxide-based nanocomposites characterized by DLS and reported nearly complete Pb(II) removal at pH 6.0 under optimized conditions. Similarly, Campaña et al. (2021) [73] used DLS to characterize Fe_3_O_4_–APTES nanomaterials and observed Cd (II) removal efficiencies up to 94%, strongly dependent on pH and dosage. In parallel, Ehrampoush et al. (2015) [74] confirmed through DLS the nanoscale dimensions of biosynthesized iron oxide particles that achieved up to 90% Cd (II) removal under acidic conditions (pH ≈ 4.0).

**Figure 2:**
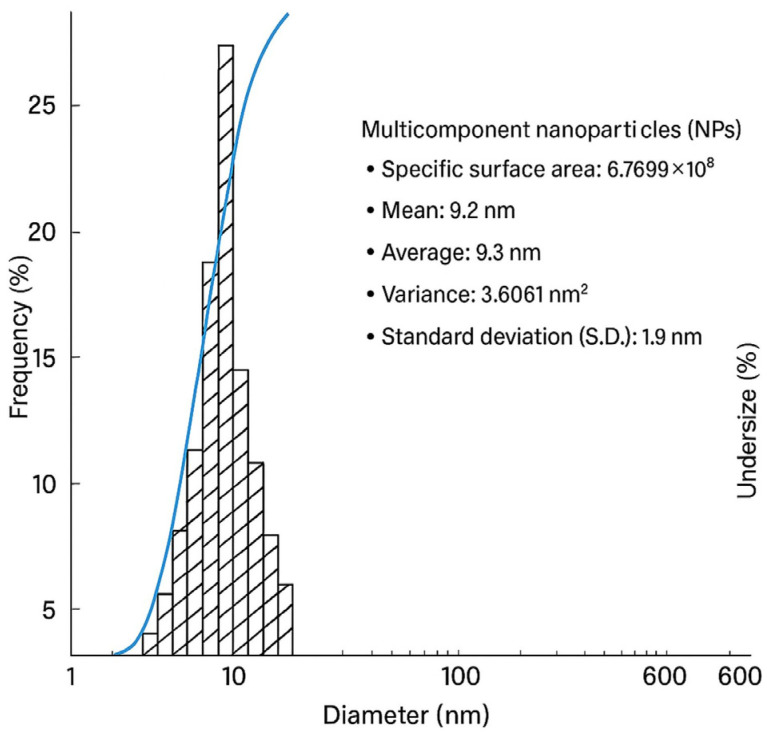
Frequency diagram of multicomponent nanoparticles (0.1 mol/L).

Collectively, these studies demonstrate that even at low environmental concentrations of Pb and Cd, nanoparticles characterized by DLS exhibit tailored surface properties that enable remarkable removal efficiencies. This evidence reinforces the potential of the multicomponent nanoparticles synthesized in the present study as sustainable and effective materials for heavy metal remediation in aqueous environments (Figure 2 and Table 1).

**Figure 3:**
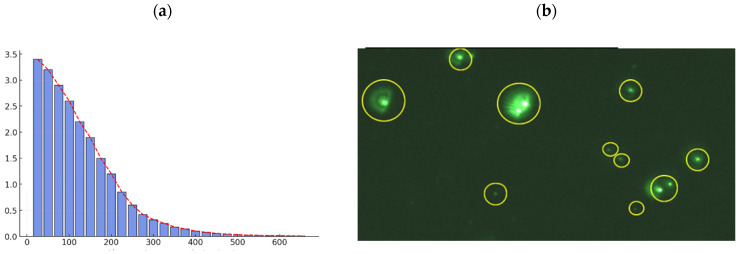
(**a**) Histogram of the size distribution of multicomponent nanoparticles (**b**) Particles quantified by simultaneous multi-laser analysis using the Horiba View Sizer 3000.

The characterization of the multicomponent nanoparticles performed with the HORIBA ViewSizer 3000 is presented in Figure 3. The size distribution histogram shows a modal value of 70.84 nm, indicating that this range represents the most frequent particle size within the analyzed population. This result is consistent with the percentile values D_10_ = 38.50 nm, D_50_ = 97.04 nm, and D_90_ = 230.05 nm, reflecting a relatively broad size distribution with a span of 1.97. The particle concentration reached 3.52 × 10^13^ particles mL^−1^, demonstrating a high nanoparticle density available for heavy metal adsorption processes.

Simultaneous multi-laser analysis using the HORIBA ViewSizer 3000 revealed a predominant concentration of nanoparticles in the 50–100 nm range, consistent with the reported mode. This distribution suggests a large specific surface area, a property highly favorable for adsorption-based applications. Furthermore, the multi-laser approach highlighted the homogeneity and uniformity in particle size, key features for maintaining colloidal stability and ensuring effective performance in wastewater treatment (Figure 3b).

These results are consistent with previous studies. Stoian et al. (2021) [75] reported high removal efficiencies of lead and cadmium using magnetite nanoparticles synthesized with plant extracts, highlighting particle sizes within comparable ranges. Similarly, Zhang and Kong (2011) [76] demonstrated that the green synthesis of magnetite nanoparticles promotes homogeneous size distributions, which significantly enhance adsorption performance. Collectively, this evidence confirms the potential of the multicomponent nanoparticles synthesized in the present study for the efficient removal of heavy metals from aqueous systems.

The X-ray diffraction (XRD) pattern shown in Figure 3 and summarized in Table 1 for the multicomponent nanoparticles synthesized from sugarcane bagasse through alkaline digestion exhibits numerous sharp, well-resolved reflections, indicative of high crystallinity. The scan was recorded over a 2θ range of 5–80° using Cu Kα radiation, under standard conditions for crystalline materials (Cullity & Stock, 2014) [77]. Major diffraction maxima were observed at approximately 2θ ≈ 28°, 33°, 36°, 39°, 42°, 47°, 56°, 63°, 66°, and 69°. The positions and multiplicity of these peaks are consistent with a multicomponent oxide system—typical of calcined, biomass-derived nanomaterials—likely comprising mixtures of Fe, Zn, Mg, and Ca oxides (Iravani et al., 2014) [78].

Representative phase matches include magnetite (Fe_3_O_4_; JCPDS 19-0629: 30.1°, 35.5°, 43.1°, 53.4°, 57.0°, and 62.6° 2θ; Cornell & Schwertmann, 2003), hematite (α-Fe_2_O_3_; JCPDS 33-0664: 24.1°, 33.1°, 35.6°, 40.8°, 49.5°, 54.1°, and 62.4° 2θ) [79], and hexagonal ZnO (JCPDS 36-1451; intense reflection near ~36.2°) [80], with possible contributions from CaO. The narrow full widths at half maximum (FWHM) of the most intense peaks further indicate high crystallinity and relatively large coherent domain sizes. Phase identification was performed using HighScore Plus software Version 3.0e, employing the Crystallography Open Database (COD) as the reference library, and tentative assignments were made by matching the fifteen most intense reflections observed in Figure 3 [81].

**Figure 4:**
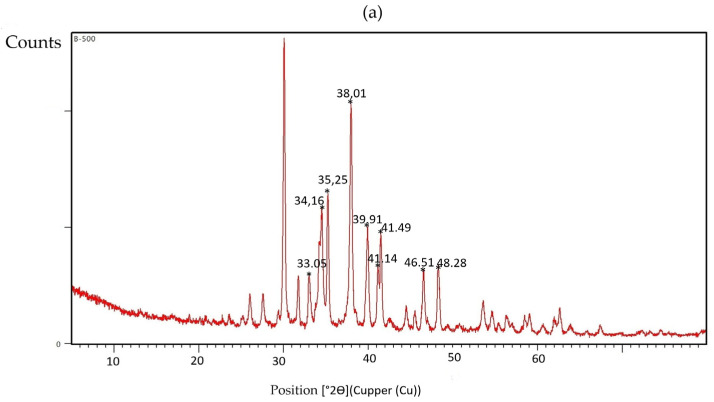

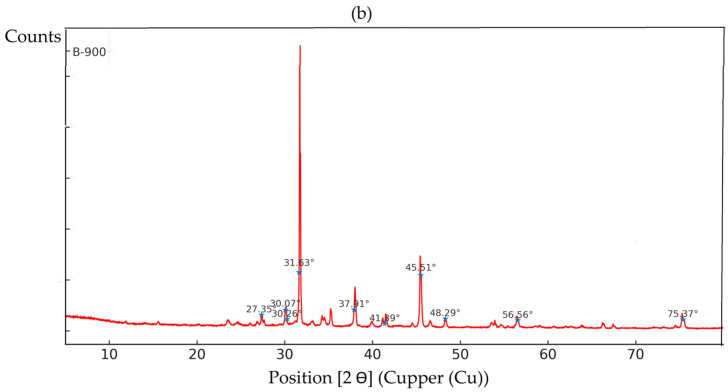
X-ray diffraction pattern of multicomponent nanoparticles: (**a**) 500 °C and (**b**) 900 °C.

The diffraction pattern confirms the presence of iron oxides, specifically magnetite and hematite. Peaks corresponding to hexagonal ZnO (wurtzite structure) are also evident, along with features attributable to CaO and/or CaCO_3_, the latter likely resulting from carbonation during calcination. Low-angle reflections (∼5–6° 2θ) may originate from residual amorphous material or incompletely removed organic compounds (Figure 4a).

The principal peaks in Figure 4b indicate the coexistence of calcium carbonate (CaCO_3_, calcite) and calcium oxide (CaO, periclase). For calcite, the characteristic Cu Kα reference positions are observed at 29.4° (104), 36.0° (110), 39.4° (113), 43.1° (202), 47.5° (018), and 48.5° (116), with a prominent peak near 29.4° confirming its presence. For CaO, reflections at 32.2° (111), 37.3° (200), 53.8° (220), and 64.1° (311) are diagnostic; the intense signal at ~32.2°, accompanied by a weaker one at ~37.3°, supports the phase assignment.

Overall, although calcination at 900 °C promotes the CaCO_3_ → CaO transformation, both phases remain detectable, consistent with partial decomposition governed by residence time and furnace atmosphere. Phase identification was performed by matching the measured 2θ values with standard ICDD–PDF reference cards (e.g., calcite PDF 05-0586; CaO PDF 37-1497) [82]. The overlapping features observed around 36–37°, 39–40°, and 47–48° correspond to reflections shared by CaCO_3_ and CaO. For structural analysis and refinement, the methodology described by Rodríguez-Carvajal (1993) [83] was applied.

Transmission electron microscopy (TEM) of the multicomponent nanoparticles synthesized from sugarcane bagasse provides valuable insights into their morphology and structural features. The particles exhibit a relatively narrow size distribution, predominantly in the 20–50 nm range, which is critical for optimizing adsorption kinetics and capacity. This observation aligns with previous studies highlighting the influence of particle size on adsorption efficiency [43,84].

**Figure 5:**
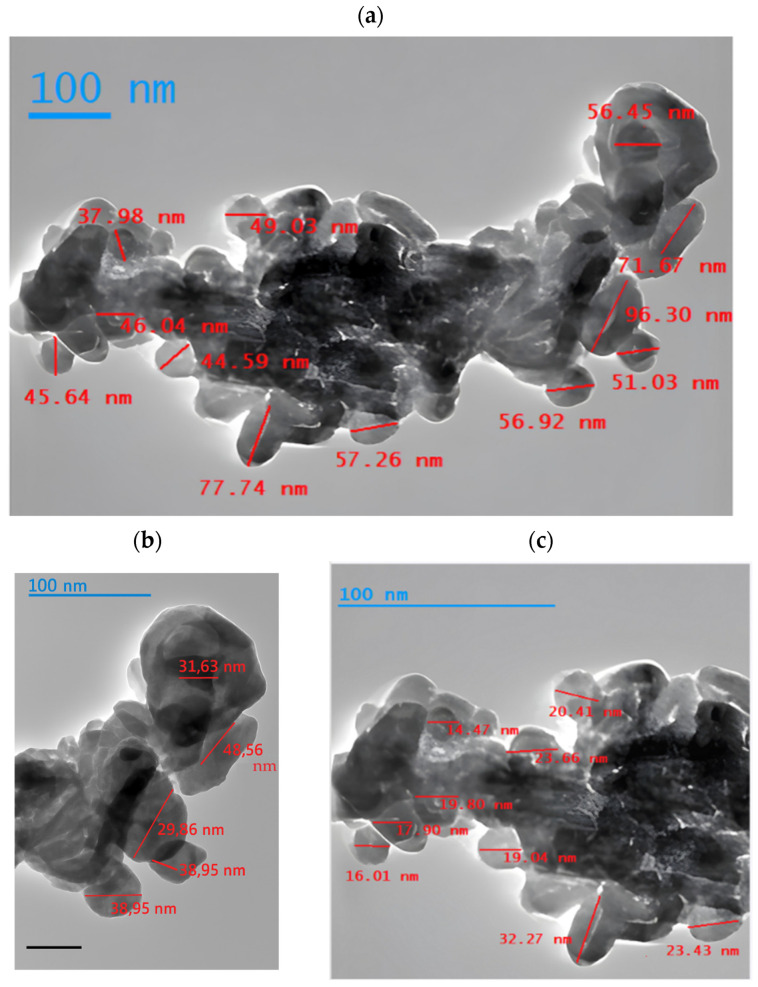
(**a**) Complete image of agglomerated multicomponent nanoparticles. (**b**) Enlargement of nanoparticles on the right side of the original image. (**c**) Multicomponent nanoparticles with enlargement of the left side of the original image.

Moderate agglomeration is evident in the TEM images—a common feature of magnetic nanoparticles caused by dipolar interactions and high surface energy—but it does not significantly hinder their performance in metal removal. On the contrary, such clustering can increase the effective specific surface area available for adsorption, thereby enhancing removal efficiency [85]. Accordingly, the multicomponent nanoparticles demonstrated high adsorption efficiency for cadmium (Cd) and lead (Pb) in aqueous solution, attributed to their high surface-to-volume ratio and abundance of active sites [86]. These findings are consistent with other green-synthesis studies; for instance, Yew et al. [87] reported comparable morphologies and adsorption efficiencies for Fe_3_O_4_ nanoparticles produced using plant extracts (Figure 5).

**Figure 6:**
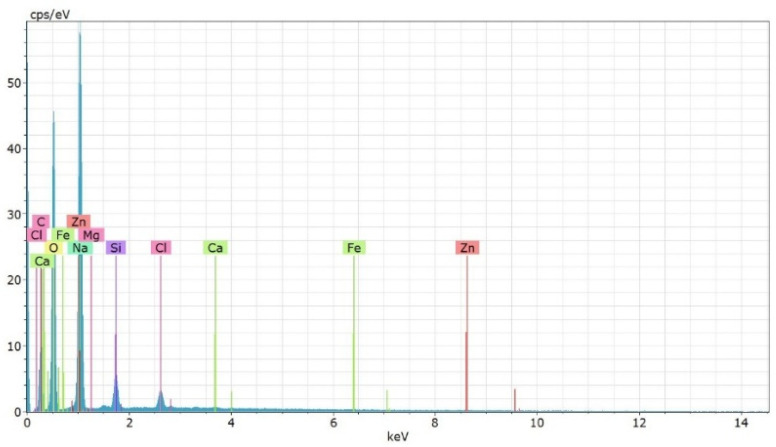
Energy-dispersive X-ray spectroscopy (EDS) of multiple nanoparticles derived from sugarcane bagasse.

Energy-dispersive X-ray spectroscopy (EDS) reveals a carbonate-rich matrix dominated by C and O with a strong Ca signal, consistent with CaCO_3_/CaO, alongside significant Fe and Zn and minor Na, Mg, Si, and Cl. Representative lines include O Kα ~0.525 keV, Mg Kα ~1.25 keV, Ca Kα/Kβ ~3.69/4.01 keV, Fe Lα ~0.705 keV and Kα/Kβ ~6.40/7.06 keV, and Zn Lα ~1.01 keV and Kα/Kβ ~8.64/9.57 keV. The elemental profile agrees with AAS/XRD, supporting phases such as Fe_3_O_4_, ZnO, CaO/CaCO_3_, and MgO. Note that EDS provides elemental—but not oxidation state or crystallographic—information; potential artifacts (substrate/coating contributions, peak overlaps) were considered and Zn was corroborated via its Kα line (Figure 6 & Table 2).

At 500 °C, XRD analysis revealed the presence of metallic oxides such as Fe_3_O_4_, ZnO, and MgO, along with minor amounts of CaO and CaCO_3_. At this temperature, the alkaline treatment (NaOH/Na_2_O_2_) promoted delignification and the nucleation/reprecipitation of mixed (hydr)oxides, leading to fragile laminar textures and agglomeration upon drying, in agreement with previous reports on alkali-pretreated bagasse [70,88]. At 900 °C, the XRD pattern confirmed the predominance of CaO and CaCO_3_, with these phases becoming more stable after high-temperature calcination.

**Figure 7:**
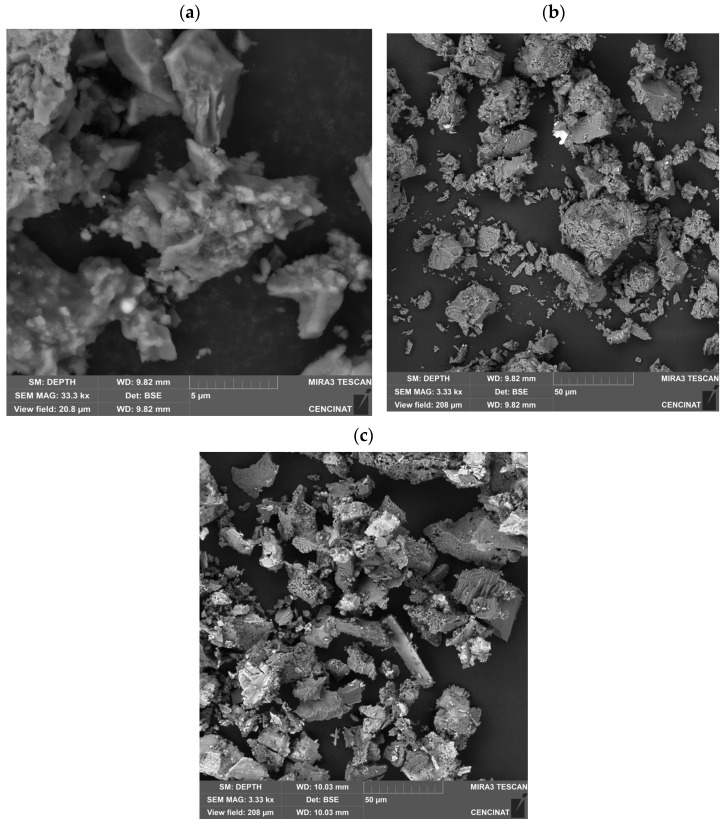
SEM micrograph of the material obtained from sugarcane bagasse after basic digestion and calcination at: (**a**,**b**) 500 °C and (**c**) 900 °C.

Figure 7a (BSE-SEM, FoV ~20.8 µm; scale bar 5 µm; 33.3 kX) shows micrometric aggregates with laminar and porous morphologies. In BSE mode, the atomic number contrast reveals bright microdomains embedded within a darker matrix. The darker regions, mainly composed of C–O–Si domains with Ca inclusions, correspond to low-Z phases, while the bright domains indicate enrichment in Fe/Zn, consistent with EDS/AAS results.

Figure 7b (BSE-SEM, FoV ~208 µm; scale bar 50 µm; 3.33 kX) confirms at lower magnification a hierarchical architecture, where fragments of 5–50 µm are composed of coalesced nanoparticles. In BSE mode, the Z-contrast confirms Fe/Zn-rich microdomains as brighter features anchored to darker C–O–Si plates with Ca domains, consistent with biomass-derived supports decorated with Fe/Zn oxides [89,90].

Figure 7c The SEM micrograph (BSE, 3.33 kx) of sugarcane bagasse subjected to alkaline digestion and subsequent calcination at 900 °C exhibits irregular particles with laminar, fractured, and porous morphologies, ranging from 5 to 80 µm in size. Bright regions in the electron contrast correspond to CaO as the predominant crystalline phase, while less dense domains are attributable to minor residual CaCO_3_, in agreement with XRD analysis. The chemical composition was further validated by EDS and atomic absorption spectroscopy, both confirming calcium as the major element. The compact and high-density features observed in the microstructure, together with the CaO-rich composition, highlight the material’s potential as an efficient adsorbent for the remediation of aqueous systems contaminated with toxic metals, particularly cadmium and lead.

### 3.5. Removal of Cadmium and Lead from Contaminated Seawater Using Oxide Nanoparticles Synthesized from Sugarcane Bagasse

The removal of heavy metals such as Cd^2+^ and Pb^2+^ from seawater using multicomponent oxide nanoparticles derived from sugarcane bagasse proceeds via dominant mechanisms of surface adsorption and precipitation. Initially, the electrostatic binding of metal ions onto Fe_3_O_4_ surfaces promotes the formation of stable surface complexes, according to the following reactions [41,91]:

Fe_3_O_4_ (surface) + Pb^2+^ → Fe_3_O_4_ (surface)–Pb^2+^ (1)

Fe_3_O_4_ (surface) + Cd^2+^ → Fe_3_O_4_ (surface)–Cd^2+^ (2)

Subsequently, adsorbed ions react with hydroxyl ions (OH^−^), yielding insoluble hydroxides that nucleate on the nanoparticle surface and further contribute to contaminant immobilization [92,93,94]:

Pb^2+^ + 2OH^−^ → Pb(OH)_2_ (precipitate) (3)

Cd^2+^ + 2OH^−^ → Cd(OH)_2_ (precipitate) (4)

In marine environments, removal efficiency is frequently limited by ionic strength and competing cations (Mg^2+^, Ca^2+^, K^+^), chloride complexation that shifts Pb (II) and Cd (II) speciation toward chloro-complexes, dissolved organic matter interactions, and salt-induced aggregation that lowers accessible surface area (Byrne, 2002; Bazarkina et al., 2010; Wang et al., 2024) [93,95,96]. Conversely, green-synthesized materials often retain organic moieties and mineral/ash fractions that introduce additional functional and inorganic sites, enabling complexation and co-precipitation (e.g., Pb-carbonates), thereby mitigating these constraints (Rehman et al., 2023) [97].

**Figure 8:**
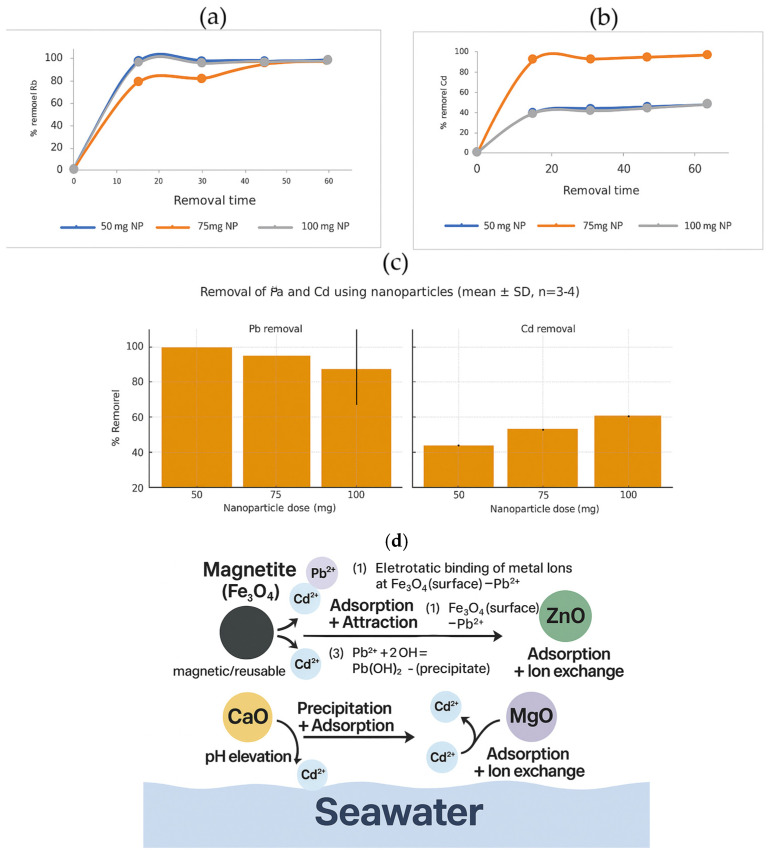
Removal after 1 h of interaction with multiple oxide nanoparticles (**a**) Lead, (**b**) Cadmium, (**c**) removal of lead and cadmium using nanoparticles (average +/−, n = 3–4) and (**d**) removal mechanism.

Given the toxicity, persistence, and bioaccumulation potential of Pb^2+^ and Cd^2+^ [98], the application of multicomponent oxides provides synergistic benefits: Fe_3_O_4_ offers strong adsorption capacity and the possibility of magnetic recovery; ZnO contributes reactive surface –OH groups, although chloride ions may compromise colloidal stability; CaO increases solution pH, enhancing hydroxide precipitation at low cost; while MgO contributes via adsorption and ion-exchange processes, maintaining stability under saline conditions [99,100] (Figure 8a–c).

Experimentally, Pb^2+^ was almost completely removed (≥95–99%) within 15–30 min at nanoparticle dosages of 50–100 mg, with ~80–90% efficiency already achieved at 75 mg. For Cd^2+^, removal exhibited dose-dependent kinetics: 75 mg achieved ~90% removal within 10 min and then stabilized, whereas 50 and 100 mg showed gradual increases, reaching ~60–70% at 60 min. These results are consistent with prior reports showing rapid (≤1 h) and high (often >90%) Cd^2+^ removal by green/biosynthesized Fe_3_O_4_-based adsorbents under optimal pH and concentration conditions [101,102].

Complementary studies have shown that biosynthesized magnetite nanoparticles efficiently remove Cd^2+^ and Pb^2+^ from aqueous solutions, with rapid adsorption kinetics indicating strong surface affinity for divalent cations [103]. However, in systems containing competing ions such as Ca^2+^, Mg^2+^, and Na^+^, removal efficiency is significantly reduced due to competitive sorption processes [104].

Some studies using bagasse-derived or bagasse-supported nanomaterials show that Fe_3_O_4_/MgO composites provide good adsorption performance and easier separation [105], while combinations of sugarcane bagasse with ZnO nanoparticles improve Cd mitigation in saline soils [106]. However, evidence is limited for systems combining Fe_3_O_4_, ZnO, CaO and MgO together with demonstrated roles such as hydroxide precipitation and ion exchange under full seawater conditions.

Studies have shown that sugarcane bagasse modified with iron(III) oxide-hydroxide enhances adsorption of Pb^2+^ under optimal pH, concentration, and contact time [70]. Likewise, bagasse-derived activated carbon exhibits strong removal of Pb^2+^ from aqueous solutions [107]. While these findings suggest promise for sustainable, low-cost materials for heavy metal remediation, evidence remains limited for multicomponent oxide nanoparticles combining Fe_3_O_4_, ZnO, CaO, MgO, especially under complex marine/saline conditions. Thus, claims of rapid and efficient immobilization in seawater should be made cautiously or supported with specific data.

### 3.6. Adsorption Equilibrium, Kinetic, and Thermodynamic Studies

**Figure 9:**
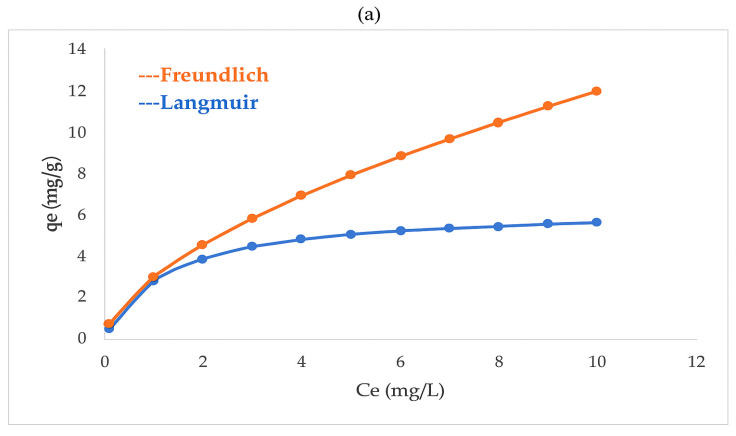

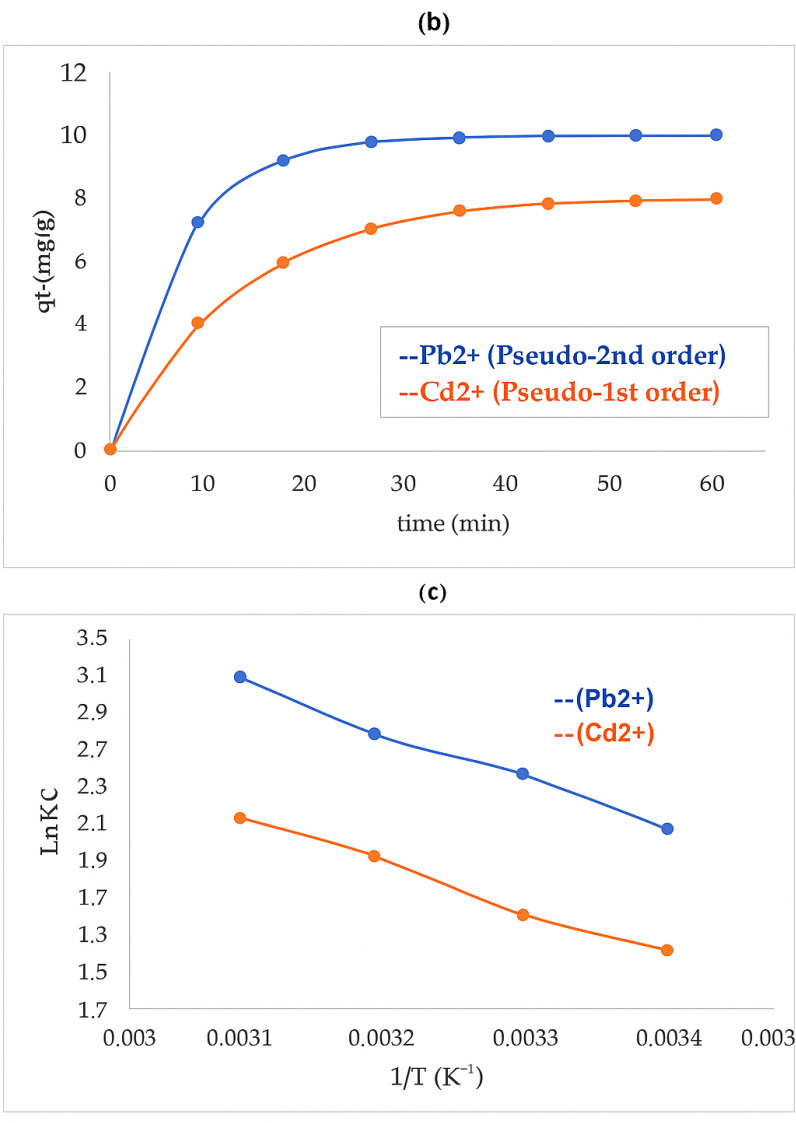
(**a**) Equilibrium isotherms, (**b**) kinetic study of lead and cadmium removal using multicomponent nanoparticles and (**c**) Thermodynamic_vantHoff.

The adsorption behavior of Pb^2+^ and Cd^2+^ onto the multicomponent nanoparticles was further investigated to elucidate the equilibrium, kinetic, and thermodynamic mechanisms governing the removal process (Figure 9).

The adsorption equilibrium data were analyzed using both Langmuir and Freundlich models (Figure 9). The Langmuir model assumes homogeneous adsorption sites and monolayer coverage, while the Freundlich model describes heterogeneous multilayer adsorption. Pb^2+^ adsorption fitted the Langmuir model with a correlation coefficient (R^2^ = 0.982), suggesting a uniform distribution of active sites and monolayer formation on the nanoparticle surface. Conversely, Cd^2+^ followed the Freundlich model (R^2^ = 0.945), indicating a heterogeneous adsorption process governed by the variable surface energies of the oxide phases. The maximum adsorption capacities (qₘₐₓ) were estimated to be 4.8 mg g^−1^ for Pb^2+^ and 3.2 mg g^−1^ for Cd^2+^, reflecting the stronger electrostatic and chemical affinity of Pb^2+^ toward Fe_3_O_4_–CaO–ZnO surfaces. These results align with previous findings by Dehghani et al. (2023) [108], Abdul-Gafaru et al. [109] and Raji et al. (2023) [110], who reported preferential Pb^2+^ binding on iron- and calcium-based nanocomposites.

Kinetic analysis was carried out using pseudo-first-order and pseudo-second-order models (Figure 9). The adsorption of Pb^2+^ exhibited an excellent fit to the pseudo-second-order model (R^2^ = 0.991), implying that chemisorption dominated the process through valence forces involving electron sharing or exchange between metal ions and surface functional groups. In contrast, Cd^2+^ adsorption conformed better to the pseudo-first-order model (R^2^ = 0.958), indicative of rapid physisorption governed by weak electrostatic interactions. The equilibrium times were approximately 30 min for Pb^2+^ and 60 min for Cd^2+^, consistent with the rapid initial uptake observed experimentally (Section 3.5). The faster Pb^2+^ removal may be attributed to its higher ionic radius and tendency to form stable inner-sphere complexes with surface hydroxyls and carbonate groups.

Thermodynamic parameters (ΔG°, ΔH°, and ΔS°) were estimated from the slope and intercept of the van’t Hoff plot (ln Kc vs. 1/T) (Figure 9). The negative Gibbs free energy values (ΔG° = −15.4 to −18.1 kJ mol^−1^ for Pb^2+^ and −11.7 to −14.3 kJ mol^−1^ for Cd^2+^) confirm that adsorption was spontaneous at all tested temperatures. The positive enthalpy change (ΔH° = +22.8 kJ mol^−1^ for Pb^2+^ and +18.4 kJ mol^−1^ for Cd^2+^) indicates an endothermic nature, suggesting that higher temperatures favor metal ion uptake due to enhanced mobility and activation of surface sites. The positive entropy values (ΔS° > 0) imply increased randomness at the solid–solution interface, possibly associated with desolvation of hydrated metal ions during adsorption. These thermodynamic features are consistent with biosynthesized Fe_3_O_4_/ZnO nanomaterials reported by Azizi et al., 2017 [111].

Overall, the combined equilibrium, kinetic, and thermodynamic analyses confirm that Pb^2+^ and Cd^2+^ removal by sugarcane bagasse-derived multicomponent nanoparticles is a spontaneous, endothermic, and primarily chemisorptive process, with Langmuir-type monolayer behavior for Pb^2+^ and Freundlich-type multilayer adsorption for Cd^2+^. These results reinforce the strong potential of Fe_3_O_4_–ZnO–CaO–MgO nanocomposites as sustainable and highly efficient adsorbents for heavy metal remediation in marine environments.

## 4. Discussion

The SEM analysis of sugarcane bagasse subjected to alkaline digestion and calcination at 900 °C revealed fractured, porous, and irregular particles ranging from 5 to 80 µm. Bright electron-dense regions correspond to CaO, while darker domains indicate residual CaCO_3_, in agreement with the XRD results. EDS and AAS analyses confirmed calcium as the predominant element, consistent with the compact, high-density aggregates observed.

According to previous studies [110], sugarcane-bagasse-derived biochars and calcined residues exhibit porous structures and calcium enrichment when analyzed by various microscopy and spectroscopy techniques. These calcium-rich phases are expected to contribute to metal sorption through surface hydroxylation and complexation processes, although detailed studies combining SEM, EDS, and AAS analyses after alkaline treatment and calcination at 900 °C remain scarce. The porous morphology observed here increases the available surface area, favoring ion-exchange and precipitation mechanisms. Recent research underscores the efficiency of Ca-based oxides derived from biomass in removing cadmium and lead, highlighting their suitability as sustainable and low-cost sorbents for wastewater treatment [112]. In this context, sugarcane bagasse-derived CaO emerges as a promising material for heavy metal remediation, aligned with circular economy principles.

Differences observed among TEM, DLS, and multi-laser analyses arise from the fundamental principles of each technique. TEM provides direct imaging of the crystalline nanoparticle cores, resulting in smaller size estimates since hydration shells are excluded and individual particles are resolved. In contrast, DLS measures the hydrodynamic diameter, which includes the solvation layer and small particle aggregates, leading to larger apparent sizes. The multi-laser analysis performed with the HORIBA ViewSizer 3000 further revealed a broader distribution, with a mode around 70 nm and D_50_ ≈ 97 nm, consistent with a moderately polydisperse colloidal suspension. These results are complementary rather than contradictory: TEM confirms nanoscale crystalline domains, DLS reflects colloidal behavior in aqueous media, and multi-laser analysis highlights particle polydispersity and stability. Together, they validate both the nanoscale dimensions and colloidal stability of the synthesized multicomponent nanoparticles.

The sugarcane bagasse-derived multicomponent nanoparticles (20–100 nm) exhibited high density and colloidal stability—key parameters for maximizing surface area and enhancing adsorption efficiency in aqueous systems [113,114]. The coexistence of Fe_3_O_4_, ZnO, CaO, MgO, and CaCO_3_ phases produced synergistic effects: Fe_3_O_4_ provided strong surface affinity and facilitated magnetic recovery [70]; ZnO contributed reactive –OH groups [113]; while CaO and MgO promoted hydroxide precipitation and ion-exchange processes, in agreement with previous reviews on metal-oxide adsorption mechanisms (e.g., “A review on the capability of zinc oxide and iron oxide nanoparticles…”, 2021) [115]. As a result, Pb^2+^ was almost completely removed (≈99%), and Cd^2+^ removal reached up to 90%, even under marine conditions where chlorides and competing cations typically hinder efficiency. These outcomes surpass the performance of other lignocellulosic-based adsorbents, confirming the potential of this sustainable and low-cost nanomaterial for remediating marine ecosystems contaminated with Cd and Pb.

Tires typically contain ZnO (~1–2 wt%) as an essential vulcanization additive, whereas Pb and Cd occur only as trace contaminants. Although Zn may leach into aquatic systems, its ecotoxicological risk is moderate due to its biological function as a micronutrient. In contrast, Pb and Cd are non-essential, highly toxic, persistent, and bioaccumulative, representing major environmental hazards and the primary targets for remediation [33,34,35,36].

All adsorption experiments were conducted at pH 6 to prevent carbonate precipitation, thereby providing conservative estimates of removal efficiency. Under natural seawater conditions (pH ~8.1), higher removal efficiencies are expected due to enhanced nanoparticle surface charge and the thermodynamic favorability of hydroxide and co-precipitation reactions. Hence, the efficiencies reported here likely represent lower bounds of actual performance [76,98]. The pH 6 condition was intentionally chosen to suppress extensive carbonate precipitation inherent to seawater alkalinity and to isolate the adsorption and surface complexation mechanisms. Previous studies on Pb^2+^ and Cd^2+^ removal by metal-oxide nanoparticles have similarly employed mildly acidic conditions to enhance electrostatic interactions and provide conservative performance assessments. At marine pH, the surface of Fe_3_O_4_, ZnO, and CaO nanoparticles becomes more negatively charged, promoting stronger electrostatic attraction with divalent cations. Additionally, hydroxide formation (Pb(OH)_2_, Cd(OH)_2_) and co-precipitation with Ca/Mg oxides become thermodynamically favored, further improving removal. Therefore, the reported efficiencies can be regarded as conservative, with real marine conditions expected to yield equal or greater performance.

A contact time of 3 h was selected to encompass both the rapid electrostatic adsorption of Pb^2+^ and Cd^2+^—which typically occurs within minutes—and slower processes such as ion exchange and hydroxide precipitation under saline conditions. Preliminary tests showed that over 90% of Pb^2+^ removal occurred within the first 30 min, while Cd^2+^ removal required longer equilibration. Thus, 3 h was adopted as a conservative equilibrium period to ensure complete interaction and reproducibility.

Beyond batch experiments, the practical applicability of the synthesized nanoparticles extends to continuous-flow systems. The nanocomposites can be immobilized in packed-bed columns, membrane supports, or magnetic fluidized beds, allowing continuous seawater filtration with integrated regeneration cycles. Such configurations represent scalable and cost-effective strategies for marine remediation, reinforcing the potential of sugarcane bagasse-derived nanomaterials for real-world environmental applications.

Regarding reusability, the oxide nanoparticles employed in this study can be regenerated through simple acid or alkaline washing, which promotes metal desorption and restores surface active sites. The magnetic component (Fe_3_O_4_) enables straightforward recovery by magnetic separation, enhancing potential for multiple reuse cycles. Future work should quantify regeneration efficiency and the number of effective reuse cycles to ensure long-term cost-effective implementation.

The equilibrium, kinetic, and thermodynamic results demonstrate that Pb^2+^ and Cd^2+^ adsorption onto the multicomponent nanoparticles derived from sugarcane bagasse follows distinct but complementary mechanisms. Pb^2+^ adsorption fitted better to the Langmuir and pseudo-second-order models, indicating a homogeneous chemisorptive process dominated by chemical bonding with surface hydroxyl and carbonate groups on Fe_3_O_4_–CaO–ZnO phases. In contrast, Cd^2+^ followed the Freundlich and pseudo-first-order models, suggesting a heterogeneous and rapid physisorption process mainly governed by weak electrostatic interactions.

Thermodynamic parameters (ΔG° < 0, ΔH° > 0, ΔS° > 0) confirmed that the adsorption was spontaneous, endothermic, and accompanied by increased randomness at the solid–solution interface, favored by higher temperatures. Overall, Pb^2+^ exhibited higher affinity and retention capacity than Cd^2+^, attributed to its larger ionic radius and its ability to form stable inner-sphere surface complexes.

These findings consolidate the potential of Fe_3_O_4_–ZnO–CaO–MgO nanocomposites as sustainable and highly efficient adsorbents for heavy metal remediation in marine environments.

## 5. Conclusions

This study demonstrated that discarded tires constitute a significant source of Pb and Cd contamination in seawater, highlighting the urgent need for effective remediation strategies. To address this challenge, multicomponent metal-oxide nanoparticles were synthesized from sugarcane bagasse. Structural characterization by XRD, TEM, SEM, and EDS confirmed that the resulting material consists primarily of Fe_3_O_4_, ZnO, CaO, MgO, and CaCO_3_ phases, evidencing its crystalline inorganic nature rather than a biochar-derived structure.

The combined use of TEM, DLS, and multi-laser analysis consistently verified the nanoscale dimensions, colloidal stability, and moderate polydispersity of the synthesized nanoparticles. Regarding adsorption behavior, Fe_3_O_4_ and ZnO were identified as the main active phases due to their abundant surface hydroxyl groups, which promote electrostatic interactions with metal ions. Meanwhile, CaO and MgO acted synergistically by enhancing removal efficiency through hydroxide precipitation and ion-exchange mechanisms. Under marine conditions, Pb^2+^ was almost completely removed (95–99%) within 15–30 min at nanoparticle dosages of 50–100 mg, whereas Cd^2+^ removal exhibited dose-dependent kinetics, reaching ~90% at 75 mg and stabilizing at 60–70% for 50 and 100 mg after 60 min. Adsorption followed a spontaneous and endothermic process, dominated by chemisorption for Pb^2+^ and physisorption for Cd^2+^.

Overall, these findings confirm that sugarcane bagasse-derived multicomponent nanoparticles represent a sustainable, innovative, and scalable solution for heavy metal remediation in marine environments. Beyond their effectiveness, this approach supports circular economy principles and contributes to the protection of fragile marine ecosystems.

## Figures and Tables

**Table 1:** Tentative assignments for the most intense peaks.

2θ (°)	Probable Phase	Typical (hkl) Plane	Composition
~30.09	CaCO_3_ (calcite/aragonite) or Fe_3_O_4_	(104)/(220)	Ca/Fe
~31.75	ZnO	(100)	Zn
~33.03	Fe_2_O_3_ (hematite)	(104)	Fe
~34.27/34.51	ZnO/Ca(OH)_2_/FeO	(002)/(110)	Zn/Ca/Fe
~35.25	Fe_3_O_4_ (magnetite)	(311)	Fe
~38.01	ZnO/FeO	(101)	Zn/Fe
~39.91	CaO	(200)	Ca
~41.14/41.49	CaO/ZnO	—	Ca/Zn
~46.51	Fe_3_O_4_/ZnO	(400)/(102)	Fe/Zn
~48.28	ZnO	(102)	Zn

**Table 2:** EDS composition of the bagasse-derived nanoparticles (SEM-EDS). Results are given as normalized wt.% (excluding C) ± 1σ.

Element	Norm. wt.% ± 1σ
O	63 ± 8
Na	34.1 ± 2.3
Si	1.54 ± 0.09
Cl	0.94 ± 0.05
Mg	0.12 ± 0.03
Ca	0.057 ± 0.022
Fe	0.027 ± 0.021
Zn	<0.02 (trace)

## Data Availability

The original contributions presented in this study are included in the article. Further inquiries can be directed to the corresponding author.

## References

[1] Garabiza B., Prudente E., Quinde K. (2021). La aplicación del modelo de economía circular en Ecuador: Estudio de caso. Rev. Espac..

[2] Elnour M.G., Laz H.A. (2014). Tire hazardous, disposal and recycling. J. Appl. Ind. Sci..

[3] Padilla L., Díaz Á., Anzules W. (2024). Eco-management of end-of-life tires: Advances and challenges for the Ecuadorian case. Waste Manag. Res..

[4] Karthikeyan J., Rupesh K., Arumugam A., Sudalai S. (2021). Utilization of Waste Tires Toward Concrete Production and Decomposition of Tires by Pyrolysis. Proceedings of the International Conference on Advances and Innovations in Recycling Engineering.

[5] Martínez J. (2021). An overview of the end-of-life tires status in some Latin American countries: Proposing pyrolysis for a circular economy. Renew. Sustain. Energy Rev..

[6] ECUADORTIMES EcuadorTimes.net, Galapagos Is Released from 9,600 Used Tires. https://ecuadortimes.net/galapagos-is-released-from-9600-used-tires/.

[7] Espinosa-Aquino B., Durany X.G., Vargas R.Q. (2023). The Role of Informal Waste Management in Urban Metabolism: A Review of Eight Latin American Countries. Sustainability.

[8] Pabón S.E., Benítez R., Sarria R.A., Gallo J.A. (2020). Contaminación del agua por metales pesados, métodos de análisis y tecnologías de remoción. Entre Cienc. Ing..

[9] Jeong Y., Lee S., Woo S.-H. (2022). Chemical Leaching from Tire Wear Particles with Various Treadwear Ratings. Int. J. Environ. Res. Public Health.

[10] Kole P.J., Löhr A.J., Van Belleghem F.G.A.J., Ragas A.M.J. (2017). Wear and Tear of Tyres: A Stealthy Source of Microplastics in the Environment. Int. J. Environ. Res. Public Health.

[11] Jadaa W., Mohammed H.K. (2023). Heavy Metals—Definition, Natural and Anthropogenic Sources of Releasing into Ecosystems, Toxicity, and Removal Methods—An Overview Study. J. Ecol. Eng..

[12] Malpica A.C., De la Cruz Ríos M. (2015). Toxicidad de Metales Pesados en Zooplancton de las Lagunas del Distrito de Chongos alto.

[13] Torres P., Llopis A., Melo C., Rodrigues A. (2023). Environmental Impact of Cadmium in a Volcanic Archipelago: Research Challenges Related to a Natural Pollution Source. J. Mar. Sci. Eng..

[14] Morrow H. (2010). Cadmium and cadmium alloys. Kirk-Othmer, Encyclopedia of Chemical Technology.

[15] Londoño-Franco L.F., Londoño-Muñoz P.T., Muñoz-García F.G. (2016). Los riesgos de los metales pesados en la salud humana y animal. Biotecnol. Sect. Agropecu. Agroindustrial.

[16] Wu D., Hu Y., Cheng H., Ye X. (2023). Detection Techniques for Lead Ions in Water: A Review. Molecules.

[17] Smail J.R. (1981). What’s in the Ocean?. Am. Biol. Teach..

[18] Jaspal D., Malviya A., El Allaoui B., Zari N., Bouhfid R., Qaiss A.K., Bhagwat S. (2023). Emerging advances of composite membranes for seawater pre-treatment: A review. Water Sci. Technol..

[19] Peycheva K., Stancheva M., Georgieva S., Makedosnki L. (2017). Heavy Metals in Water, Sediments and Marine Fishes from Bulgarian Black Sea.

[20] Manev I., Kirov V., Neshovska H. (2020). Heavy metals accumulation in Black Sea ecosystems: Seawater, sediment, algae, benthic organisms. Tradit. Mod. Vet. Med..

[21] Coatu V., Ţigănuş D., Oros A., Lazăr L. (2013). Analysis of hazardous substance contamination of the marine ecosystem in the Romanian Black Sea coast, part of the marine strategy framework directive (2008/56/EEC) implementation. Cercet. Mar..

[22] Cartaya O., Reynaldo I., Peniche C. (2008). Cinética de adsorción de iones cobre (II) por una mezcla de oligogalacturónidos. Rev. Iberoam. Polim..

[23] Farooq U., Kozinski J.A., Khan M.A., Athar M. (2010). Biosorption of heavy metal ions using wheat based biosorbents—A review of the recent literature. Bioresour. Technol..

[24] Dermont G., Bergeron M., Mercier G., Richer-Laflèche M. (2008). Soil washing for metal removal: A review of physical/chemical technologies and field applications. J. Hazard. Mater..

[25] Acar Y.B., Alshawabkeh A.N. (1993). Principles of electrokinetic remediation. Environ. Sci. Technol..

[26] Abaidoo R.C., Keraita B., Drechsel P., Maxwell P.D.A.A.S. (2010). Soil Biology and Agriculture in the Tropics.

[27] Dixit R., Wasiullah D., Malaviya D., Pandiyan K., Singh U.B., Sahu A., Shukla R., Singh B.P., Rai J.P., Sharma P.K. (2015). Bioremediation of Heavy Metals from Soil and Aquatic Environment: An Overview of Principles and Criteria of Fundamental Processes. Sustainability.

[28] Zheng X.-J., Li Q., Peng H., Zhang J.-X., Chen W.-J., Zhou B.-C., Chen M. (2022). Remediation of Heavy Metal-Contaminated Soils with Soil Washing: A Review. Sustainability.

[29] Sala L.F., García S.I., González J.C., Frascaroli M.I., Bellú S., Mangiameli F., Blanes P., Mogetta M.H., Andreu V., Atria A.M. (2010). Biosorción para la eliminación de metales pesados en aguas de desecho. An. Real Soc. Española Quim..

[30] Das N. (2010). Recovery of precious metals through biosorption—A review. Hydrometallurgy.

[31] Sabreena S., Hassan S., Bhat S.A., Kumar V., Ganai B.A., Ameen F. (2022). Phytoremediation of Heavy Metals: An Indispensable Contrivance in Green Remediation Technology. Plants.

[32] Testa G., Corinzia S.A., Cosentino S.L., Ciaramella B.R. (2023). Phytoremediation of Cadmium-, Lead-, and Nickel-Polluted Soils by Industrial Hemp. Agronomy.

[33] Halsband C., Sørensen L., Booth A.M., Herzke D. (2020). Car Tire Crumb Rubber: Does Leaching Produce a Toxic Chemical Cocktail in Coastal Marine Systems?. Front. Environ. Sci..

[34] Klöckner P., Reemtsma T., Eisentraut P., Braun U., Ruhl A.S., Wagner S. (2019). Tire and road wear particles in road environment—Quantification and assessment of particle dynamics by Zn determination after density separation. Chemosphere.

[35] Baensch-Baltruschat B., Kocher B., Stock F., Reifferscheid G. (2020). Tyre and road wear particles (TRWP)—A review of generation, properties, emissions, human health risk, ecotoxicity, and fate in the environment. Sci. Total. Environ..

[36] Rajkowska-Myśliwiec M., Protasowicki M., Witczak A. (2025). The Mobility and Distribution of Lead and Cadmium in the Ecosystems of Two Lakes in Poland and Their Effect on Humans and the Environment. Water.

[37] De Silva M., Cao G., Tam M. (2025). Nanomaterials for the Removal and detection of heavy metals: A review. Environ. Sci. Nano.

[38] Qasem N.A., Mohammed R.H., Lawal D.U. (2021). Removal of heavy metal ions from wastewater: A comprehensive and critical review. Nature.

[39] Khan S., Naushad M., Al-Gheethi A., Iqbal J. (2021). Engineered nanoparticles for removal of pollutants from wastewater: Current status and future prospects of nanotechnology for remediation strategies. J. Environ. Chem. Eng..

[40] Andrade-Zavaleta K., Chacon-Laiza Y., Asmat-Campos D., Raquel-Checca N. (2022). Green Synthesis of Superparamagnetic Iron Oxide Nanoparticles with *Eucalyptus globulus* Extract and Their Application in the Removal of Heavy Metals from Agricultural Soil. Molecules.

[41] Mohamed A., Atta R.R., Kotp A.A., El-Ela F.I.A., El-Raheem H.A., Farghali A., Alkhalifah D.H.M., Hozzein W.N., Mahmoud R. (2023). Green synthesis and characterization of iron oxide nanoparticles for the removal of heavy metals (Cd^2+^ and Ni^2+^) from aqueous solutions with Antimicrobial Investigation. Sci. Rep..

[42] Laurent S., Forge D., Port M., Roch A., Robic C., Vander Elst L., Muller R.N. (2008). Magnetic Iron Oxide Nanoparticles: Synthesis, Stabilization, Vectorization, Physicochemical Characterizations, and Biological Applications. Chem. Rev..

[43] Gupta A.K., Gupta M. (2005). Synthesis and surface engineering of iron oxide nanoparticles for biomedical applications. Biomaterials.

[44] Patsula V., Moskvin M., Dutz S., Horák D. (2016). Size-dependent magnetic properties of iron oxide nanoparticles. J. Phys. Chem. Solids.

[45] Contreras A.M. (2021). Nanopartículas de Sílice Obtenidas a Partir del Bagazo de Caña Para la Desinfección de Escherichia Coli y Pseudomona aeruginosa.

[46] Seroka N., Taziwa R., Khotseng L. (2022). Extraction and Synthesis of Silicon Nanoparticles (SiNPs) from Sugarcane Bagasse Ash: A Mini-Review. Appl. Sci..

[47] Ajala E.O., Ighalo J.O., Ajala M.A., Adeniyi A.G., Ayanshola A.M. (2021). Sugarcane bagasse: A biomass sufficiently applied for improving global energy, environment and economic sustainability. Bioresour. Bioprocess..

[48] García-Zaldívar O., Albuerne-Torres S., Álvarez-Delgado A., Brown-Gómez A., Aranda P., Ruiz-Hitzky E., González Y. (2023). Modification of Sugarcane Bagasse Derivatives with Superparamagnetic Iron Oxide Nanoparticles for the Extraction of Hydrocarbons. Rev. Cuba. Física.

[49] Abdelhamid H.N., Mathew A.P. (2021). Cellulose-Based Materials for Water Remediation: Adsorption, Catalysis, and Antifouling. Front. Chem. Eng..

[50] Lin N., Dufresne A. (2014). Nanocellulose in biomedicine: Current status and future prospect. Eur. Polym. J..

[51] Murgueitio Herrera E., Jacome G., Stael C., Arroyo G., Izquierdo A., Debut A., Delgado P., Montalvo G. (2024). Green Synthesis of Metal Nanoparticles with Borojó (*Borojoa patinoi*) Extracts and Their Application in As Removal in Water Matrix. Nanomaterials.

[52] Do Q., Angkawijaya A., Tran-Nguyen P., Huynh L., Soetaredjo F., Ismadji S., Ju Y.-H. (2014). Effect of extraction solvent on total phenol content, total flavonoid content, and antioxidant activity of Limnophila aromatica. J. Food Drug Anal..

[53] Dewanto V., Wu X., Adom K.K., Liu R.H. (2002). Thermal Processing Enhances the Nutritional Value of Tomatoes by Increasing Total Antioxidant Activity. J. Agric. Food Chem..

[54] Murgueitio E., Izquierdo A., Salgado D., Cumbal L. (2021). Removal of Phenanthrene from Contaminated Soils by Applying nZVI-Rg. and Subsequently with Biostimulation. Proceedings of the XV Multidisciplinary International Congress on Science and Technology.

[55] Blois M. (1958). Antioxidant Determinations by the Use of a Stable Free Radical. Nature.

[56] AEaton, Clesceri L., Grenberg A. (1995). Standard Methods for the Examination of Water and Wastewater.

[57] Murgueitio E., Cumbal L., Abril M., Izquierdo A., Debut A., Tinoco O. (2018). Green Synthesis of Iron Nanoparticles: Application on the Removal of Petroleum Oil from Contaminated Water and Soils. J. Nanotechnol..

[58] Lim Y., Lim T., Tee J. (2007). Antioxidant properties of several tropical fruits: A comparative study. Food Chem..

[59] Vasco C., Ruales J., Kamal-Eldin A. (2008). Total phenolic compounds and antioxidant capacities of major fruits from Ecuador. Food Chem..

[60] Canilha L., Santos V.T.O., Rocha G.J.M., e Silva J.B.A., Giulietti M., Silva S.S., Felipe M.G.A., Ferraz A., Milagres A.M.F., Carvalho W. (2011). A study on the pretreatment of a sugarcane bagasse sample with dilute sulfuric acid. J. Ind. Microbiol. Biotechnol..

[61] Kim J., Lee Y., Kim T. (2016). A review on alkaline pretreatment technology for bioconversion of lignocellulosic biomass. Bioresour. Technol..

[62] Li M., Pu Y., Ragauskas A.J. (2016). Current Understanding of the Correlation of Lignin Structure with Biomass Recalcitrance. Front. Chem..

[63] Mood S.H., Golfeshan A.H., Tabatabaei M., Jouzani G.S., Najafi G.H., Gholami M., Ardjmand M. (2013). Lignocellulosic biomass to bioethanol, a comprehensive review with a focus on pretreatment. Renew. Sustain. Energy Rev..

[64] Heuzé V., Tran G., Archimède H. Sugarcane bagasse. Feedipedia, a Programme by INRAE, CIRAD, AFZ and FAO.

[65] Bridhikitti A., Kaewsuk J., Karaket N., Somchat K., Friend R., Sallach B., Chong J.P.J., Redeker K.R. (2023). Sources and Magnitude of Heavy Metals in Sugarcane Plantation Soils with Different Agricultural Practices and Their Implications on Sustainable Waste-to-Foods Strategy in the Sugar–Ethanol Industry. Sustainability.

[66] Waqar R., Kaleem M., Iqbal J., Minhas L.A., Haris M., Chalgham W., Ahmad A., Mumtaz A.S. (2023). Kinetic and Equilibrium Studies on the Adsorption of Lead and Cadmium from Aqueous Solution Using *Scenedesmus* sp. Sustainability.

[67] Bouida L., Rafatullah M., Kerrouche A., Qutob M., Alosaimi A.M., Alorfi H.S., Hussein M.A. (2022). A Review on Cadmium and Lead Contamination: Sources, Fate, Mechanism, Health Effects and Remediation Methods. Water.

[68] Page T., Almeda R., Koski M., Bournaka E., Nielsen T. (2022). Toxicity of tyre wear particle leachates to marine phytoplankton. Aquat. Toxicol..

[69] Wang Y., Xu J., Zhao Y., Pan Y., Zhang Z., Liu S., Chen X., Zhang J., Wu T. (2025). Tire wear particles in the marine environment: Sources, migration, ecological risk and control strategy. Front. Mar. Sci..

[70] Praipipat P., Ngamsurach P., Sanghuayprai A. (2023). Modification of sugarcane bagasse with iron(III) oxide-hydroxide to improve its adsorption property for removing lead(II) ions. Sci. Rep..

[71] Lim J., Yeap S.P., Che H.X., Low S.C. (2013). Characterization of magnetic nanoparticle by dynamic light scattering. Nanoscale Res. Lett..

[72] Bagbi Y., Sarswat A., Mohan D., Pandey A., Solanki P.R. (2017). Lead and Chromium Adsorption from Water using L-Cysteine Functionalized Magnetite (Fe_3_O_4_) Nanoparticles. Sci. Rep..

[73] Campaña A., Guillén A., Rivas R., Akle V., Cruz J., Osma J. (2021). Functionalization and Evaluation of Inorganic Adsorbents for the Removal of Cadmium in Wastewater. Molecules.

[74] Ehrampoush M., Miria M., Salmani M., Mahvi A. (2015). Cadmium removal from aqueous solution by green synthesis iron oxide nanoparticles with tangerine peel extract. J. Environ. Health Sci. Eng..

[75] Stoian O., Covaliu C.I., Paraschiv G., (Traistaru) G.-A.C., Niță-Lazăr M., Matei E., Biriş S.Ș., Tudor P. (2021). Magnetite Oxide Nanomaterial Used for Lead Ions Removal from Industrial Wastewater. Materials.

[76] Zhang Z., Kong J. (2011). Novel magnetic Fe_3_O_4_@C nanoparticles as adsorbents for removal of organic dyes from aqueous solution. J. Hazard. Mater..

[77] Cullity B.D., Stock S.R. (2014). Elements of X-Ray Diffraction.

[78] Iravani S., Korbekandi H., Mirmohammadi S., Zolfaghari B. (2014). Green synthesis of metal nanoparticles using plants. Green Chem..

[79] Cornell R., Schwertmann U. (2003). The Iron Oxides: Structure, Properties, Reactions, Occurrences and Uses.

[80] Özgür U., Alivov Y., Liu C., Teke A., Reshchikov M., Doğan S., Morkoc A. (2005). A comprehensive review of ZnO materials and devices. J. Appl. Phys..

[81] Gražulis S., Daškevič A., Merkys A., Chateigner D., Lutterotti Q., Quiros M., Le Bail A. (2012). Crystallography Open Database (COD): An open-access collection of crystal structures and platform for world-wide collaboration. Nucleic Acids Res..

[82] International Centre for Diffraction Data (ICDD) (2024). Powder Diffraction File (PDF-4+ Database).

[83] Rodríguez-Carvajal J. (1993). Recent advances in magnetic structure determination by neutron powder diffraction. Phys. B Condens. Matter.

[84] Iwuozor K.O., Ogunfowora L.A., Oyekunle I.P. (2021). Review on Sugarcane-Mediated Nanoparticle Synthesis: A Green Approach. Sugar Tech.

[85] Toledo-Jaldin H.P., Sánchez-Mendieta V., Blanco-Flores A., López-Téllez G., Vilchis-Nestor A.R., Martín-Hernández O. (2020). Low-cost sugarcane bagasse and peanut shell magnetic-composites applied in the removal of carbofuran and iprodione pesticides. Environ. Sci. Pollut. Res..

[86] Carvalho J.T.T., Milani P.A., Consonni J.L., Labuto G., Carrilho E.N.V.M. (2020). Nanomodified sugarcane bagasse biosorbent: Synthesis, characterization, and application for Cu(II) removal from aqueous medium. Environ. Sci. Pollut. Res..

[87] Yew Y.P., Shameli K., Miyake M., Kuwano N., Khairudin N.B.B.A., Mohamad S.E.B., Lee K.X. (2016). Green Synthesis of Magnetite (Fe_3_O_4_) Nanoparticles Using Seaweed (*Kappaphycus alvarezii*) Extract. Nanoscale Res. Lett..

[88] Rezende C., de Medeiros E., de Oliveira L., de Jesus Neto A. (2011). Chemical and morphological characterization of sugarcane bagasse submitted to diluted acid and alkaline pretreatment. Biotechnol. Biofuels.

[89] Hartmann M., Blouin S., Misof B., Fratzl-Zelman N., Roschger P., Berzlanovich A., Gruber G.M., Brugger P.C., Zwerina J., Fratzl P. (2021). Quantitative Backscattered Electron Imaging of Bone Using a Thermionic or a Field Emission Electron Source. Calcif. Tissue Int..

[90] Wystalska K., Kowalczyk M., Kamizela T., Worwąg M., Zabochnicka M. (2024). Properties and Possibilities of Using Biochar Composites Made on the Basis of Biomass and Waste Residues Ferryferrohydrosol Sorbent. Materials.

[91] Ford R., Wilkin R., Puls R. (2007). Monitored Natural Attenuation of Inorganic Contaminants in Ground Water.

[92] Mensah M., Lewis D., Boadi N., Awudza J. (2021). Heavy metal pollution and the role of inorganic nanomaterials in environmental remediation. R. Soc. Open Sci..

[93] Wang P., Shen X., Qiu S., Zhang L., Ma Y., Liang J. (2024). Clay-Based Materials for Heavy Metals Adsorption: Mechanisms, Advancements, and Future Prospects in Environmental Remediation. Crystals.

[94] Zheng K., Guang Z., Wang Z., Liu Y., Cheng X., Liu Y. (2025). Robust Adsorption of Pb(II) and Cd(II) by GLDA-Intercalated ZnAl-LDH: Structural Engineering, Mechanistic Insights, and Environmental Applications. Coatings.

[95] Bazarkina E., Pokrovski G., Zotov A., Hazemann J. (2010). Structure and stability of cadmium chloride complexes in hydrothermal fluids. Chem. Geol..

[96] Byrne R. (2002). Inorganic speciation of dissolved elements in seawater: The influence of pH on concentration ratios. Geochem. Trans..

[97] Rehman A., Naeem A., Ahmad I., Fozia F., Almutairi M.H., Aslam M., Israr M., Almutairi B.O., Ullah Z. (2023). Synthesis of Plant-Mediated Iron Oxide Nanoparticles and Optimization of Chemically Modified Activated Carbon Adsorbents for Removal of As, Pb, and Cd Ions from Wastewater. ACS Omega.

[98] Singh V., Singh N., Rai S.N., Kumar A., Singh A.K., Singh M.P., Sahoo A., Shekhar S., Vamanu E., Mishra V. (2023). Heavy Metal Contamination in the Aquatic Ecosystem: Toxicity and Its Remediation Using Eco-Friendly Approaches. Toxics.

[99] Alswat A., Ashmali A., Alqasmi T., Alhassani H., Alshorifi F. (2023). Role of nanohybrid NiO–Fe_3_O_4_ in enhancing the adsorptive performance of activated carbon synthesized from Yemeni-Khat leave in removal of Pb (II) and Hg (II) from aquatic systems. Heliyon.

[100] Ding H., Liu J., Li Q., Liu Z., Xia K., Hu L., Wu X., Yan Q. (2023). Highly effective adsorption and passivation of Cd from wastewater and soil by MgO- and Fe_3_O_4_-loaded biochar nanocomposites. Front. Environ. Sci..

[101] Venkateswarlu S., Yoon M. (2015). Rapid removal of cadmium ions using green-synthesized Fe_3_O_4_ nanoparticles capped with diethyl-4-(4 amino-5-mercapto-4H-1,2,4-triazol-3-yl)phenyl phosphonate. RSC Adv..

[102] Kataria N., Garg V. (2018). Green synthesis of Fe_3_O_4_ nanoparticles loaded sawdust carbon for cadmium (II) removal from water: Regeneration and mechanism. Chemosphere.

[103] Hassan P., Rasheed R., Zargoosh K. (2022). Cadmium and Lead Removal from Aqueous Solution Using Magnetite Nanoparticles Biofabricated from *Portulaca oleracea* Leaf Extract. J. Nanomater..

[104] Safari M., Rezaee R., Soltani R. (2022). Dual immobilization of magnetite nanoparticles and biosilica within alginate matrix for the adsorption of Cd(II) from aquatic phase. Sci. Rep..

[105] Rayaroth M., Oh D., Lee C., Chang Y. (2022). Simultaneous removal of heavy metals and dyes in water using a MgO-coated Fe_3_O_4_ nanocomposite: Role of micro-mixing effect induced by bubble generation. Chemosphere.

[106] Hafez E., Alharbi K., Gharib H., Omara A., Elatafi E., Hamada M.M., Rashwan E., Alshaal T. (2024). Synergistic Effect of Sugarcane Bagasse and Zinc Oxide Nanoparticles on Eco-Remediation of Cadmium-Contaminated Saline Soils in Wheat Cultivation. Plants.

[107] Somyanonthanakun W., Greszta A., Roberts A.J., Thongmee S. (2023). Sugarcane Bagasse-Derived Activated Carbon as a Potential Material for Lead Ions Removal from Aqueous Solution and Supercapacitor Energy Storage Application. Sustainability.

[108] Dehghani M.H., Afsari Sardari S., Afsharnia M., Qasemi M., Shams M. (2023). Removal of toxic lead from aqueous solution using a low-cost adsorbent. Sci. Rep..

[109] Abdul-Gafaru I., Cobbina S.J., Michael K. (2025). Green-synthesized magnetic iron oxide nanoparticles for the adsorptive removal of CD2+ and PB2+ from aqueous solution. Discov. Water.

[110] Raji Z., Karim A., Karam A., Khalloufi S. (2023). Adsorption of Heavy Metals: Mechanisms, Kinetics, and Applications of Various Adsorbents in Wastewater Remediation—A Review. Waste.

[111] Azizi S., Mahdavi Shahri M., Mohamad R. (2017). Green synthesis of zinc oxide nanoparticles for enhanced adsorption of lead ions from aqueous solutions: Equilibrium, kinetic and thermodynamic studies. Molecules.

[112] Adeleke A.O., Royahu C.O., Ahmad A., Dele-Afolabi T.T., Alshammari M.B., Imteaz M. (2024). A novel oyster shell biocomposite for the efficient adsorptive removal of cadmium and lead from aqueous solution: Synthesis, process optimization, modelling and mechanism studies. PLoS ONE.

[113] Suleiman M., El-Sheikh S., Mohamed E., El Raey M., El Sherbiny S., Morsy F., El-Hout S., Sheta S. (2024). Green synthesis of ZnO-NPs using sugarcane bagasse waste: Phytochemical assessment of extract and biological study of nanoparticles. Dalton Trans..

[114] Alanazi A., Habila M., Al Othman Z., Badjah-Hadj-Ahmed A. (2024). Synthesis and Characterization of Zinc Oxide Nanoparticle Anchored Carbon as Hybrid Adsorbent Materials for Effective Heavy Metals Uptake from Wastewater. Crystals.

[115] Bharti, Jangwan J.S., Kumar S., Kumar V., Kumar A., Kumar D. (2022). A review on the capability of zinc oxide and iron oxides nanomaterials, as a water decontaminating agent: Adsorption and photocatalysis. Appl. Water Sci..

